# Evaluation of the Cardiac Electrophysiological and Haemodynamic Effects of *Elsholtzia ciliata* Essential Oil on Swine

**DOI:** 10.3390/ph15080982

**Published:** 2022-08-10

**Authors:** Vilma Zigmantaitė, Eglė Jonušaitė, Ramunė Grigalevičiūtė, Audrius Kučinskas, Rimantas Treinys, Antanas Navalinskas, Vaidotas Žvikas, Valdas Jakštas, Lauryna Pudžiuvelytė, Jurga Bernatonienė, Regina Mačianskienė, Jonas Jurevičius

**Affiliations:** 1 Biological Research Center, Lithuanian University of Health Sciences, Tilžės St. 18/7, LT47181 Kaunas, Lithuania; 2 Laboratory of Membrane Biophysics, Institute of Cardiology, Lithuanian University of Health Sciences, Sukilėlių Ave. 15, LT50162 Kaunas, Lithuania; 3 Institute of Pharmaceutical Technologies, Faculty of Pharmacy, Lithuanian University of Health Sciences, Sukilėlių Ave. 13, LT50162 Kaunas, Lithuania; 4 Laboratory of Biopharmaceutical Research, Institute of Pharmaceutical Technologies, Faculty of Pharmacy, Medical Academy, Lithuanian University of Health Sciences, Sukilėlių Ave. 13, LT50162 Kaunas, Lithuania; 5 Department of Drug Technology and Social Pharmacy, Medical Academy, Lithuanian University of Health Sciences, Sukilėlių Ave. 13, LT50162 Kaunas, Lithuania

**Keywords:** antiarrhythmic effect, electrophysiological effect, *Elsholtzia ciliata* (Thunb.) Hyl., essential oil, herbal medicine, haemodynamic effect, hypotensive effect, *Sus scrofa domesticus*

## Abstract

The demand for the development of novel medicines with few side effects and no proarrhythmic properties is increasing. Extensive research on herbal extracts has been conducted with the expectation that the compounds will exert precise effects without harmful side effects. *Elsholtzia ciliata* (Thunb.) Hyl. essential oil (*EO*) possesses antiarrhythmic properties similar to those of class 1B antiarrhythmics, such as prolonging myocardial activation of the QRS complex and shortening the QT interval. In this study, we determined the kinetic profile of *EO* phytocompounds and the effects of *EO* on heart electrical activity and arterial blood pressure. For this study, we chose to use local breed pigs that were anaesthetized. The effects of an intravenous bolus of *EO* on ECG parameters, arterial blood pressure, heart rate variability, and blood levels of haematological and biochemical parameters were registered and evaluated. Following an intravenous injection of a bolus, *EO* exerted a vasodilatory effect, resulting in significant reductions in arterial blood pressure. EO also increased the heart rate and altered ECG parameters. The bolus of *EO* prolonged the QRS complex, shortened the QT interval, and nonmonotonically altered the PQ interval. After the administration of a bolus of *EO*, the activity of the autonomic nervous system was altered. This study confirms that *EO* possesses similar properties to class 1B antiarrhythmics and exerts a hypotensive effect; it reduces arterial blood pressure possibly by modulating peripheral vascular resistance.

## 1. Introduction

Modern Western society is affected by an increasing number of cardiovascular diseases, including arrhythmias, ischaemic heart disease, and high blood pressure [1,2,3]. Cardiac arrhythmias remain a leading cause of morbidity and mortality in developed countries [4]. According to the World Health Organization and other sources, more than 40% of people over the age of 25 years live with high blood pressure. The highest prevalence in underdeveloped or developing countries is due to malnutrition and a lack of medicines [5]. Notably, current pharmaceuticals are not perfect due to proarrhythmic side effects and have not been shown to reduce mortality in large randomized and placebo-controlled studies [6,7,8]. Herbal medications might be an alternative remedy for cardiovascular diseases due to minimal side effects and their ability to exert a wide range of pharmacological effects by targeting various physiological processes.

Essential oils are volatile liquids that contain many chemical compounds, such as oxygenated monoterpenes, monoterpene hydrocarbons, oxygenated sesquiterpenes, and sesquiterpene hydrocarbons [9,10]. Essential oils possess antibacterial [11,12,13,14], antioxidant [15,16], anti-inflammatory [17], antiviral, and antifungal [18] activities. According to scientific data, essential oils reduce stress [19], inhibit cancer cell growth [20], decrease pain [21], and promote wound healing [22]. The flowering plant *Elsholtzia ciliata* (Thunb.) Hylander of the family Lamiaceae is native to Asia and is also found in Africa, North America, Europe, and India [23]. *E. ciliata* is widely used in folk medicine due to its antibacterial, anticancer, and anti-inflammatory properties [24].

*E. ciliata* essential oil (*EO*) consists of approximately twenty organic components (see Table S1 [25]). Previously, our team identified and patented the antiarrhythmic effect of the extract from the whole plant of *E. ciliata* [26,27] in the isolated heart; because the whole organism is a perfectly balanced system with protective, compensatory, and activating mechanisms, herbal extracts must be tested in an animal model in vivo. In this study, we investigated the effects on not only the heart, but also the vascular system. The aim of this study was to investigate the antiarrhythmic effect of*EO* and to evaluate its effects on the cardiovascular and haemodynamic systems in vivo in the anaesthetized pig.

## 2. Results

### 2.1. Effect of Elsholtzia ciliata on ECG Parameters

**Figure 1:**
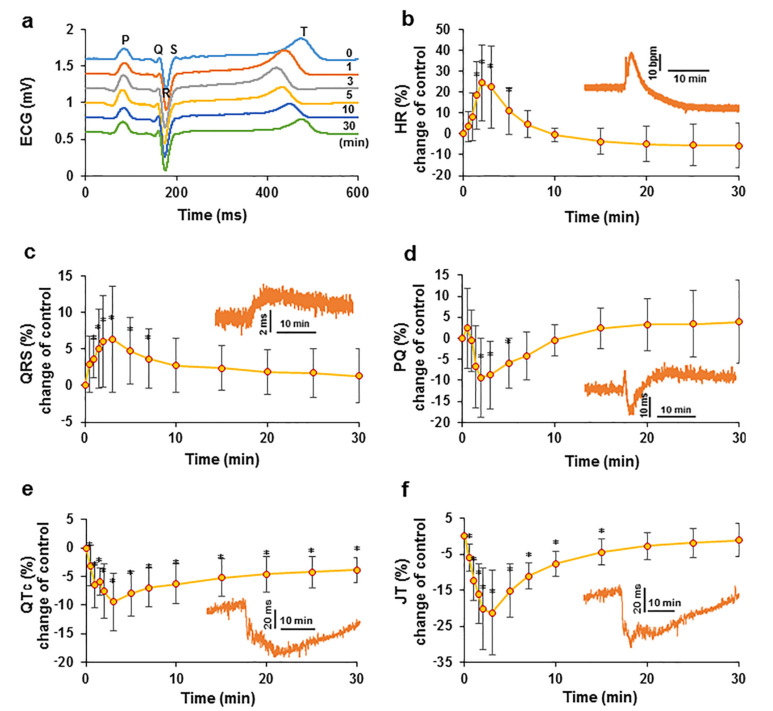
Effect of an intravenous injection of an *EO* bolus on cardiac electrical activity in swine. Changes in electrocardiogram parameters over time compared to the control: cardiac cycle ECG recordings at different times after the administration of the *EO* bolus (**a**); HR (**b**); QRS complex (**c**); PQ interval (**d**); QTc interval (**e**) and JT interval (**f**). The data are presented as percent changes compared to the control. The insets show data from a representative experiment. * *p*  <  0.05 indicates a significant difference from controls, *n* = 8.

After an intravenous injection of a bolus of *EO* (30 µL/kg), ECG parameters were recorded and subsequently evaluated to characterize the effect of the herbal substance on the electrophysiological functions of different parts of the heart. Data on ECG parameters are presented at times consistent with the analysis of the kinetic profile of *EO* phytocompounds (see the Methods), except in special cases. ECG recordings at different times are presented in Figure 1a.

The biphasic effect of *EO* on heart rate (HR) was observed in the study. After the intravenous injection of the bolus, HR increased (*p* < 0.05) immediately to 114.82 ± 23.11 bpm at T2 compared with a control value of 92.02 ± 12.00 bpm. Thereafter, HR started to decrease and approached the control value of 91.41 ± 11.28 bpm at T10, followed by a further decrease, and the HR was 86.83 ± 15.16 bpm at T30 (Figure 1b).

The QRS complex reflects the propagation of electrical impulses in the ventricles. After an intravenous injection of the bolus of *EC*, the QRS complex became significantly prolonged (*p* < 0.05) at T3, reaching a maximum value of 0.0565 ± 0.0099 s compared with the control value of 0.0531 ± 0.0083 s. After T7, the QRS complex gradually shortened but remained nonsignificantly longer than that of the control (Figure 1c).

The PQ interval mainly reflects atrioventricular node passage, i.e., the time it takes for an electrical impulse to travel from the sinus node across the atrioventricular node until it enters the ventricles. This parameter is measured on ECG recordings from the beginning of the P wave to the Q wave. In the present study, following an intravenous injection of a bolus of *EO*, a transient increase was observed in the PQ interval at T0.5 to a value of 0.1030 ± 0.0013 s from a control value of 0.1006 ± 0.0088 s, followed by a decrease to the minimum value of 0.0907 ± 0.0082 s at T2. The PQ interval was then lengthened and approached the control value at T10; after this transient shortening, the PQ interval increased to a value higher than that of the control. At T30, it was nonsignificantly longer than that of the control (Figure 1d).

The change in the QTc interval during the study was the only parameter for which statistically significant changes compared with the control (*p* < 0.05) were observed throughout the acquisition period of 30 min. After an intravenous injection of the *EO* bolus, the QTc interval showed a significant nonmonotonic decrease. The minimum QTc interval duration was 0.3849 ± 0.04510 s at T3, which was shorter than the control measurement of 0.4270 ± 0.0603 s. After this time point, the interval gradually increased but remained significantly lower than that of the control (Figure 1e).

The JT interval represents the repolarization process. After an intravenous injection of the *EO* bolus, the JT interval was significantly shortened (*p* < 0.05) from the control value of 0.2922 ± 0.0462 s to its minimum value of 0.2268 ± 0.0299 s at T3, followed by a gradual return to a level close to the control value (Figure 1f).

The average data for other ECG parameters are presented in the supplements. The RR interval decreased significantly after an intravenous injection of an *EO* bolus (Figure S1a). The amplitude of the T wave varied nonmonotonically but remained elevated compared with that of the control (Figure S1b). The kinetics of the QT interval duration after *EO* bolus administration are presented in the supplements (Figure S1c).

### 2.2. Effect of EO on Arterial Blood Pressure

**Figure 2:**
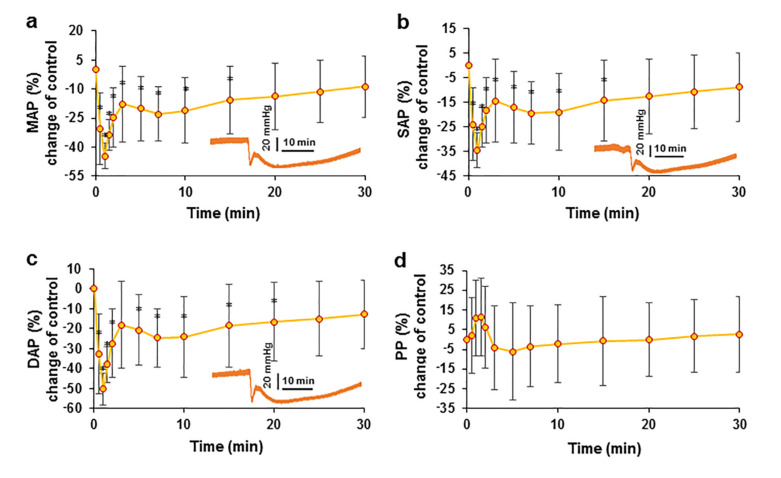
Effect of an intravenous injection of an *EO* bolus on arterial blood pressure in pigs. The changes in MAP (**a**), SAP (**b**), DAP (**c**), and PP (**d**) after intravenous bolus administration are presented as percent changes compared with the control. * *p* <  0.05 indicates a significant difference from controls, *n* = 8.

Following an intravenous injection of an *EO* bolus, a significant reduction in arterial blood pressure (*p* < 0.05) was observed. The change in mean arterial blood pressure (MAP) after intravenous injection of the bolus after normalization to the control is shown in Figure 2a. From a control value of 80.65 ± 14.97 mmHg, MAP decreased immediately after the bolus was administered to its lowest value of 44.39 ± 8.06 mmHg recorded at T1. Thereafter, an increase in arterial blood pressure was observed; at T3, MAP increased to 65.62 ± 15.34 mmHg, and a delayed lower hypotensive effect of 62.91 ± 19.45 mmHg was subsequently measured at T7. The hypotensive effect decreased thereafter, but MAP remained lower than that of the control throughout the experiments.

A similar change in arterial blood pressure kinetics was observed when measuring systolic (SAP) (Figure 2b) and diastolic (DAP) (Figure 2c) arterial blood pressure. The change in pulse pressure (PP) was non-significant, but it should be noted that PP was elevated during the first two minutes (Figure 2d). This initial increase in PP may indicate that the initial decrease in DAP was greater than that in SAP.

### 2.3. Evaluation of the Relationship between Blood Pressure and HR after the Administration of the EO Bolus

**Figure 3:**
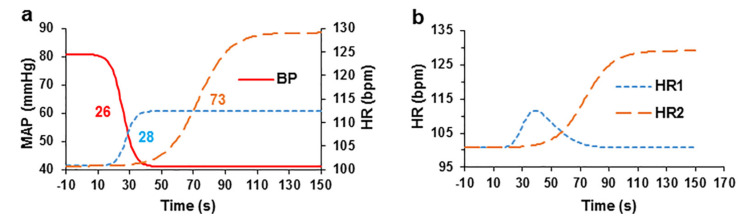
Interdependence of blood pressure and HR after intravenous administration of the *EO* bolus in pigs. (**a**) The relationship between blood pressure and HR components after administration of the *EO* bolus (all curves were fitted with sigmoidal logistic curves). The numbers next to the curves indicate the half-lives from the start of administration of the EC bolus. (**b**) The curves for the two HR components that were fitted with an asymmetric Gaussian curve (first component, HR1) and with a sigmoidal logistic curve (second component, HR2), *n* = 4.

We analysed the kinetics of changes in blood pressure and HR during the initial exposure after *EO* bolus administration to evaluate the relationship between blood pressure and the HR. Figure 3 shows the blood pressure and HR kinetic curves. The values of these parameters were recorded every 5 s within an interval of 1 s. The hypotensive effect of the *EO* bolus on MAP occurred earlier than the increase in the HR.

During analysis of the data for each experiment, logistic sigmoidal curves were used to approximate the decrease in blood pressure and increases in two HR increase and to calculate their half-lives t_1/2_. The t_1/2_ obtained from the curves of the increases in HR increase were compared with the t_1/2_ of the sigmoidal curve of the decrease in arterial blood pressure to assess their onset of action. Mathematical subtraction of the first component of the increased HR was performed by approximating it with an asymmetric Gaussian curve to calculate the second phase of the increase in HR. The first component of the increase in HR was not observed in all experiments; however, in 4 experiments, it was sufficiently large to estimate the data by approximation with the sigmoidal curve.

The sigmoidal curve showing decreased MAP with a t_1/2_ of 25.58 ± 5.90 s and slope of −0.275 ± 0.095 was calculated. The two components of the increase in HR are presented in two separate sigmoidal logistic curves. The initial transient phase of the curve showing increased HR had a t_1/2_ of 27.60 ± 4.85 s and slope of 0.339 ± 0.072, and the second curve showing a delayed increase in HR had a t_1/2_ of 73.01 ± 12.05 s and slope of 0.102 ± 0.032.

### 2.4. Effects of EO on Heart Rate Variability

**Figure 4:**
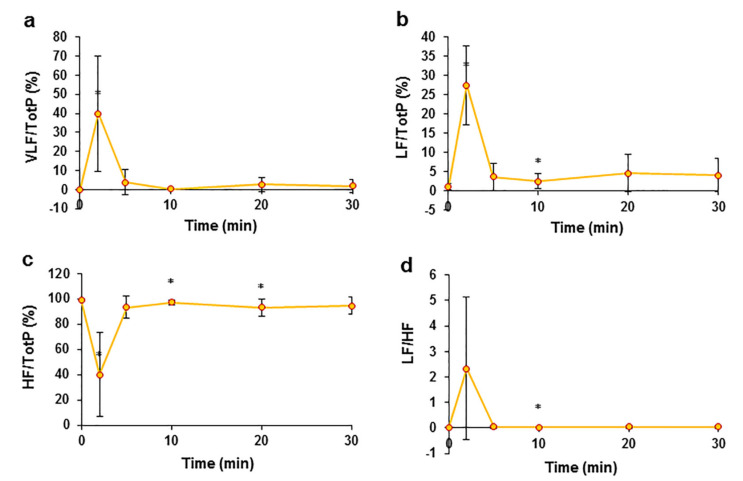
The effects of an intravenous injection of an *EO* bolus on the frequency domain of pig HRV. Ratio of very low frequency power (VLF) to total power of frequency (Tot P) (**a**), changes in the ratio of the LF component to total frequency power (**b**), changes in the ratio of HF to total frequency power changes after the administration of the *EO* bolus (**c**), and LF/HF (**d**). * *p* < 0.05 indicates at significant difference compared to the control, *n* = 8.

Heart rate variability (HRV) was analysed in the study to evaluate the changes in autonomic nervous system activity. After an intravenous injection of an *EO* bolus, a sudden change in all frequency domains of HRV parameters was registered (Figure 4), with maximal changes observed at T2; after this point, all registered parameters returned almost to the control values. Moreover, after the administration of the *EO* bolus, the ratio of low frequency (LF) power to total power was reported as the percent change (Figure 4b) from the control measurement and ranged from 1.05 ± 0.90% to 27.50 ± 10.23% at T2. The opposite change was observed in the ratio of high frequency (HF) to total power (Figure 4c) measurements from the control and was 98.83 ± 0.93%, which decreased to 40.07 ± 33.17%. After bolus-induced changes, the low frequency-to-high frequency ratio (LF/HF) increased from the control value of 0.01 ± 0.01 to 2.33 ± 2.79 (Figure 4d). These data show a clear change in the activity of the autonomic nervous system after the administration of the EO bolus. The values of time-domain parameters and their changes after bolus administration are presented in the supplements (Table S2).

### 2.5. Kinetic Profile of EO Phytochemicals after Intravenous Bolus

**Figure 5:**
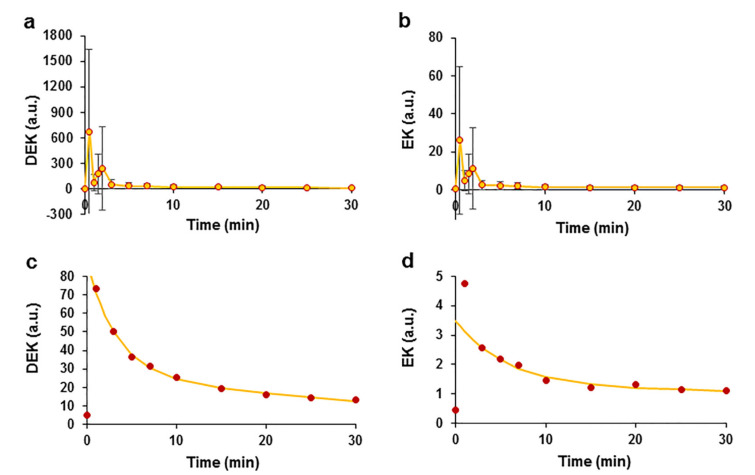
Pharmacokinetics of the *E. ciliata* ketones DEK and EK after intravenous administration of the *EO* bolus in swine. Time courses of DEK (**a**) and EK (**b**) in auxiliary units. The kinetics of decreases in the DEK (**c**) and EK (**d**) concentrations were approximated using the double exponential function (lines) (the same data as in (**a**,**b**) are shown at higher magnification), (circles)—experimental points indicate concentrations in a.u. (**a**–**d**), *n* = 8.

*EO*, which consists of more than 20 phytocompounds (Figure S1), with the main compounds identified as the ketones DEK and EK, was used in the study. We analysed the kinetic profile of the ketones. The plots of plasma concentration against time showed peaks for DEK and EK at retention times (RTs) of 3.61 min for DEK and 3.74 min for EK. Plasma concentrations were calculated by estimating the peak area, and the mean data from 8 experiments are reported in auxiliary units (Figure 5). After summing the results, two peaks that occurred at the T0.5 and T2 time points were registered. The maximum value of DEK was 674.86 ± 970.13 a.u. measured at time point T0.5 (Figure 5a). Similar kinetics of EK with a maximum value of 25.98 ± 38.82 a.u. were measured at T0.5 (Figure 5b). A monotonic decrease in the concentrations of both ketones was observed beginning at time point T3. This decrease in concentrations at time points ranging from T3 to T30 was estimated by averaging the data and using a double exponential function, with time constants of 3.2 min and 35.2 min for DEK (Figure 5c) and 5.8 min and 303.6 min for EK (Figure 5d).

**Figure 6:**
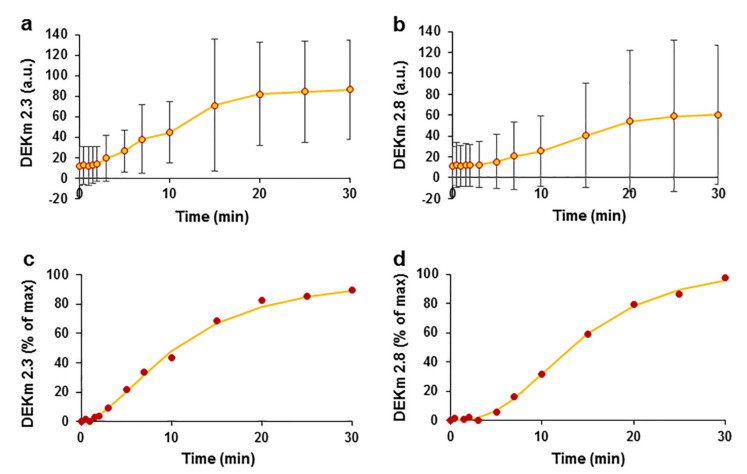
Kinetics of *E. ciliata* DEK metabolite after an intravenous administration of the *EO* bolus in swine. Kinetics of the DEK metabolites with RTs of 2.3 min (**a**) and 2.8 min (**b**), (circles) experimental data points reported in a.u. Kinetics of DEK metabolites at RTs of 2.3 min (**c**) and 2.8 min (**d**), normalized and average data were fitted using the Hill time response function (lines). *n* = 8.

The variation in the double exponential curves indicated that two processes that decrease the concentrations occurred that might be related to ketone elimination and metabolism. One explanation for the decrease in the parental ketone concentrations may be their efficient metabolism in the liver, similar to that of other furan ring compounds. A spectral analysis of the ketones revealed the appearance of new peaks with different retention times. During the analysis of DEK, additional peaks appeared at retention times of 2.3 min and 2.8 min, possibly representing DEK metabolites. We observed a time-dependent increase in the peak amplitude of DEK metabolites (Figure 6a,b). The average data for DEK metabolites were normalized to the maximum value and were approximated using the Hill equation, which is a time response function. The metabolite at an RT of 2.3 min had the following Hill equation parameters: t_1/2_ = 10.8 min and Hill coefficient η = 1.8 (Figure 6c); the metabolite at RT 2.8 min had a t_1/2_ = 14.03 min and η = 2.6 (Figure 6d). The correlation coefficients characterizing the quality of the fit of the Hill equation to the experimental data were R^2^ = 0.997 and R^2^ = 0.999 for the metabolites that occurred at RTs of 2.3 min and 2.8 min, respectively. Logistic sigmoidal curves of the data were also obtained but had smaller correlation coefficients of 0.985 and 0.995, respectively, compared with the Hill curve; the data are not shown.

Similarly, a new peak at an RT of 1.3 min was observed in the pharmacokinetic analysis of EK, although its amplitude was small and close to the measurement resolution, and these data were not analysed and are not shown.

### 2.6. The Effect of EO on Blood Parameters

Blood levels of haematological and biochemical parameters were examined during the study. All studied parameters and their values are presented as the means ± SEM in Table S3, and most of the studied parameters changed nonsignificantly. The white blood cell (WBC) counts decreased significantly (*p* < 0.05) to 10.23 ± 2.11 10^9^ cells L^−^^1^ and 10.38 ± 2.82 10^9^ cells L^−^^1^ at T1 and T5, respectively, from the control value of 12.24 ± 2.47 10^9^ cells L^−^^1^. Moreover, a significant decrease in the platelet (PLT) count was observed immediately after the injection (T1) from the control value of 318.25 ± 75.97 10^9^ platelets L^−^^1^ to 290.38 ± 76.02 10^9^ platelets L^−^^1^.

During the study, the EO bolus significantly (*p* < 0.05) increased the blood potassium concentration from the control K^+^ value of 3.70 ± 0.34 mmol/L to 3.88 ± 0.44 mmol/L, 3.90 ± 0.42 mmol/L, and 3.93 ± 0.44 mmol/L at T1, T5, and T30, respectively. Throughout the experiment, the K^+^ level tended to increase but did not exceed the normal value in pigs. Changes in blood gas parameters after EO administration were evaluated in venous blood.

## 3. Discussion

Eight local pig breeds were used in this study to measure the pharmacological effects of *EO*. The effects on electrophysiological parameters and arterial blood pressure were registered and evaluated in anaesthetized animals to eliminate spontaneous respiratory movements and other disturbances that might occur in conscious animals. The arterial blood pressure data measured in the study correspond to pigs of the same age and weight [28]. Our research has shown that this remedy has electrophysiological features characteristic for antiarrhythmic agent that also appears to act on blood vessels, reducing blood pressure in pigs. Further preclinical and human studies are needed to determine whether this herbal substance is an effective treatment for cardiac arrhythmias and hypertension.

The kinetic profile showed a rapid and transient increase in *EO* ketone concentrations at time points ranging from 30 s to 3 min in various experiments, followed by an exponential monotonic decrease in ketone concentrations, which was well estimated using a dual exponential function, possibly due to the elimination and metabolism of the parental ketones. A rapid transient increase in ketone concentrations is possibly associated with the rapid 10-s intravenous administration of the bolus. *EO* compounds are highly lipophilic, and these compounds may interact with cells in the blood or organs in various systems and thus participate in pharmacological processes [29,30].

The correlation between the in vivo results and the *EO* chemical composition might be attributed to the main volatile compounds—EK and DEK. After an initial increase, monotonic decreases in DEK and EK concentrations were noticed, suggesting that both ketones from *EO* were possibly rapidly eliminated from the blood and/or metabolized in the liver, similar to other substances with furan rings in their structures [31]. New compounds detected at RTs of 1.3 min, 2.3 min, and 2.8 min that were possible metabolites of the parent ketones were observed in pharmacokinetic studies. The kinetics of metabolite formation suggest that the decreases in the concentrations of parental ketones are at least partially due to metabolism. The metabolites are expected to retain the functional activity of the parental ketones.

A comparison between obtained retention index (RI) values of *E. ciliata* compounds was compared with RIs reported in the NIST Chemistry WebBook and published research articles [32]. The authors determined the RI values of EK (RI-1199), DEK (RI-1277), germacrene D (RI-1480), α-farnesene (RI-1503), and caryophyllene oxide (RI-1583) in *E. ciliata* [33]. Other authors obtained values for isocaryophyllene (RI-1298), eugenol (RI-1216), (RI-1199), caryophyllene oxide (RI-1336), and palmitic acid (RI-1286) in EC [24]. Some RIs of volatile compounds are different, which might be due to the use of slightly different GC–MS systems and methods.

We used *EO*, which has been shown to exert antiarrhythmic effects in previous studies [26]; therefore, in vivo research revealed additional properties of this herbal substance that might be very beneficial. In this study, we analysed the effects of *EO* during the first 30 min, and thus only short-term regulation of the cardiovascular system was recorded. In this study, we attempted to evaluate the mechanism underlying the effect of *EO* on cardiac and vascular changes and the organism response to these changes. The effects of autonomic nervous system (ANS) alterations might compensate for the rapid decrease in blood pressure.

The administration of an intravenous bolus of *EO* to pigs in vivo revealed changes in ECG parameters and a hypotensive effect, and the relationship between these processes was clarified chronologically. In the present study, after the intravenous injection of the *EO* bolus, a decrease in arterial blood pressure was observed and was approximated using a sigmoidal logistic curve with a t_1/2_ of 25.58 s. This process was followed by a transient primary increase in HR (HR1) with a t_1/2_ of 27.6 s, possibly due to the ANS response to a decrease in blood pressure. We suggest that the temporal difference between the decrease in blood pressure and the increase in HR would be appropriate to generate a response through baroreceptors [34,35,36,37]. The second component of the increase in HR (HR2) was then observed with a t_1/2_ of 73.01 s. The occurrence of the second component of the increase in HR is probably explained by a slower delayed response from the cardiovascular centre to the adrenal medulla, where catecholamines (noradrenaline and adrenaline) are released, which activate the heart and increase HR [38,39].

Activation of the sympathetic nervous system is reflected in not only changes in HR but also ECG parameters in the PQ range [40], which mainly represents conduction across the atrioventricular node. The changes in the PQ interval recorded in our study are multiphasic, with a very rapid increase in the PQ interval after the intravenous bolus, possibly resulting from a direct effect of *EO* on atrioventricular node activity by slowing its conduction through blockade of the L-Type Ca^2+^ current. A similar effect has been reported in isolated rabbit hearts after exposure to higher concentrations of *EO* [26]. The effects of changes in the PQ interval are shown in Figure 3b and the insert from the representative experiment. Subsequently, the conductance in the atrioventricular node is accelerated, which may be an effect of the sympathetic nervous system (SNS) activity. The initial increase in the PQ interval may provide evidence that the decrease in blood pressure recorded in our study may be due to direct calcium channel blockade in vascular smooth muscle cells.

In addition to the direct effects of *EO* on the heart, we observed changes in cardiac function that may be related to changes in the activity of the ANS caused by baroreflexes due to a decrease in blood pressure. We measured and evaluated HRV in the present study to evaluate the effects of *EO* on the ANS. *EO* altered the activity of the ANS through mechanisms that are possibly related to an increase in the dominance of the SNS [40,41,42,43]. Higher sympathetic tone and activity are also reflected in the increased LF/HF ratio of HRV and the predominance of the LF component of the HRV frequency domain [38,44,45]. Finally, we suggest that after the administration of an *EO* bolus, the activity of the ANS is altered, possibly due to an increase in the dominant role of sympathetic tone as a secondary reflection of a decrease in arterial blood pressure. Most antihypertensive drugs have been found to alter the ANS and improve sympathetic function in patients by increasing catecholamine (noradrenaline and adrenaline) production [46,47,48], presumably through baroreflex-mediated sympathetic activation [49]. Notably, very few researchers have examined the HRV in pigs in detail and provided clear explanations of its changes; therefore, we propose that our HRV data might be used to assess the importance of changes in the pig ANS in regulating the cardiovascular system.

The visible effect of an intravenous injection of an *EO* bolus on electrocardiogram parameters may suggest that the EO modulates the electrical activity of the heart and possibly blocks Na^+^ currents and alters Ca^2+^ and K^+^ channels, resulting in prolongation of the QRS complex, shortening of QT interval, and modulation of PQ intervals. The results of this study and previous studies with an isolated rabbit heart [26] may indicate that *EO* possess similar properties to class 1B antiarrhythmics [50] that alter the electrophysiological properties of the heart primarily by blocking the first derivative of the upstroke of cardiac action potential. This is good, but not a direct measure of the Na^+^ transmembrane current and the prolonging of the QRS complex. In our study, *EO* altered other ECG parameters, which may be related to changes in Ca^2+^ and K^+^ transmembrane currents. The initial increase in the PQ interval and long-lasting shortening of the QTc interval might be explained by the inhibitory effect of *EO* on the L-type Ca^2+^ current, which slowed the propagation of electrical impulses in the atrioventricular node [51] and shortened action potentials in the ventricular myocardium. Apparently, the shortening of the QTc interval is caused not only by blocking calcium channels but also by activating potassium channels [52]. The present study showed a significant increase in blood potassium levels after *EO* administration and an increase in T wave amplitude on ECG, which supports the assumption of increased activity of K^+^ currents [53]. Elevated potassium levels may also support the hypothesis that hyperpolarizing potassium currents reduce the influx and level of calcium in vascular wall cells, leading to vascular relaxation and lower blood pressure [53,54]. However, this mechanism is not clear from the data of this study only. Further studies to investigate and elucidate this mechanism using isolated cardiac myocytes are needed. 

In the present study, during the first two minutes after the EO bolus a greater decrease in diastolic blood pressure was observed than in systolic blood pressure, and it may indicate that EO directly reduces systemic vascular or total peripheral resistance by acting on peripheral blood vessels [5,55]. This effect may be due to a direct blockade of the calcium channels of smooth muscle cells of arterial vessels, as well as other mechanisms regulating Ca^2+^ entry that are involved in reducing the membrane potential, such as activation of K+ transmembrane currents [56]. The data additionally show a decrease in the action potential duration in isolated rabbit hearts exposed to low EO concentrations [26], possibly due to increased potassium channel activity, as no modulation of atrioventricular node conduction was observed after treatment with such low doses of EO. Thus, action potential shortening at low EO concentrations could not be explained by direct blocking of Ca^2+^ channels, but already shortened action potentials can, by themselves, reduce the inward Ca^2+^ current.

We revealed that *EO* reduces the number of leukocytes, which was confirmed by previous in vitro studies in which the researchers noted that the components of *EO* had anti-inflammatory properties. All *Elsholtzia ciliata* essential oils tested in other studies showed anti-inflammatory properties, from both stem and flower. Essential oils most effectively suppressed TNF-α and IL-6-related pathways. Leaf essential oils, which blocked PGE-2 secretion to the greatest extent also exerted anticancer, antifungal, antimicrobial, and nontoxic effects on cells in cell cultures [25,57,58]. 

*E. ciliata* consists of approximately twenty organic components and at the highest proportions are the two parent ketones: dehydroelsholtzia ketone (DEK; 78.15%) and elsholtzia ketone (EK; 14.12%). According to the literature, no studies have reported the pharmacological effects of EK and DK on the heart and blood vessels. Additionally, large numbers of sesquiterpenes, such as β-bourbonene, isocaryophyllene, β-cubebene, ledene, α-caryophyllene, α-cubebene, germacrene D, trans-α-bergamotene, α-farnesene, γ-cadinene, δ-cadinene, and caryophyllene oxide are also present in *EO*. The cardiac properties of sesquiterpenes presented in *EO* have not been previously reported in the literature. Sesquiterpenes such as jatamansone isolated from the rhizomes of *Nardostachys jatamansi* showed antiarrhythmic and anticonvulsant activities [59]. Farnesol exerts biological effects, including cardioprotective, antioxidant, and antiarrhythmic properties [60]. Nerolidol attenuated myocardial infarction in an isoproterenol-treated rat model and exerted a significant effect on protecting the heart by maintaining endogenous antioxidant enzyme activities [61].

## 4. Materials and Methods

### 4.1. Animals

All animal usage, care, housing, and procedures were performed in accordance with the EU Directive 2010/63/EU on guidelines for the protection of animals used for scientific purposes and were approved (Permission No. G2-171) by the Lithuanian Commission on the Ethics of the Use of Experimental Animals at the State Food and Veterinary Services. The animal studies are reported in compliance with the ARRIVE guidelines [62]. Animals were maintained in the Lithuanian University of Health Sciences (LUHS) Biological Research Centre facility approved by the State Food and Veterinary Services Animal Welfare Department.

Lithuanian local breed pigs (*n* = 8) weighing 32 ± 1.9 kg were used in the experiments conducted in autumn 2021. Pigs were housed in the LUHS Biological Research Centre under 12/12 h light/dark cycles, were fed ad libitum, and had free access to water. During the procedure, animals were premedicated by administering intramuscular injections of xylazine hydrochloride 3 mg/kg (Sedaxylan, Eurovet Animal Health, Bladel, The Netherlands), ketamine hydrochloride 20 mg/kg (Ketamidor, Richter Pharma, Wels, Austria), and fentanyl citrate 3 µg/kg (Fentanyl-Kalceks, Riga, Latvia). Pigs were anaesthetized by intravenously injected 8 mg/kg thiopental sodium (Thiopental VUAB, Czech Republic) and intubated using a 6-in tracheal cuffed tube (tracheal tube, Intersurgical, London, UK). During all procedures, analgesia was infused continuously using a pump (Draeger, Lübeck, Germany). General anaesthesia was maintained via inhalation of sevoflurane (Sevoflo, Abbot, Abbot Park, IL, USA). All experimental tests started 40 min after intubation to avoid the cardiovascular effects of anaesthetic drugs. Anaesthesia was monitored every 5 min during the procedure, and the records are stored at the Biological Research Centre. An arterial catheter (16 G 2.2 mm × 20 cm, Arrow, Telflex, Wayne, PA, USA) was inserted into the right carotid artery with a modified Seldinger technique. The same technique was used to place a central venous catheter (18 G; 2.2 mm × 20 cm, Arrow, Telflex, Wayne, PA, USA) into the internal jugular vein.

### 4.2. Materials

*E. ciliata* herbs were collected in August 2021, and the *EO* extract was prepared prior to experiments using previously published methods [25]. The *EO* was obtained by hydro distillation from the dried grass of *E. ciliata* (i.e., during flowering, the whole aboveground part of the plant—Flowers, leaves, and stems, but not the roots were collected). A stock solution of *E. ciliata* (1:1 in ethyl alcohol, anhydrous, ≥99.8%) was freshly prepared prior to each experiment. Three experiments were performed under similar conditions using identical amounts of ethanol. The sodium chloride solution used to prepare the test solutions in a 1:1 ratio to determine the effects of intravenous alcohol on blood pressure and cardiac electrophysiological parameters, and the results did not reveal visible effects on ECG and blood pressure parameters (data not shown).

### 4.3. Experimental Protocol

A dose of 30 µL/kg *EO* was injected intravenously into the ear vein as a bolus using a syringe, and the injection lasted 10 s and was controlled by hand.

### 4.4. Time of Measurements

First, baseline ECG and invasive arterial blood pressure (IBP) recordings were performed 40 min before the experiment, and the parameters were monitored and recorded using a Siemens SC 7000 vital parameter monitor (Siemens AG, Munich, Germany). Arterial blood pressure (IBP and noninvasive (NIBP)), pulse, oximetry, and temperature were monitored using a Draeger Vista 120 (Draeger, Lübeck, Germany). Blood samples for the plasma analysis were collected through a venous catheter in the following order: T0 (zero time) or control before bolus, T0.5, T1, T1.5, T2, T3, T5, T7, T10, T15, T20, T25, and T30, with the numbers indicating the minutes after the start of the bolus injection. Blood samples for blood haematology (Mythic 18 Vet, Woodley, UK) and blood gas analyses using an EPOC blood analysis system and BGEM test cards (EPOC, Woodley, SK, Canada) were collected in the following order: T0, T1, T5, and T30.

### 4.5. Measured Parameters

Vital signs were monitored (Draeger Vista 120, Draeger, Lübeck, Germany) to obtain pulse oximetry, capnography, IBP, and NIBP measurements. IBP was measured in the carotid artery using a Siemens SC 7000 instrument (Siemens AG, Germany). Electrophysiological parameters were measured using Power Lab (ADInstruments, Dunedin, New Zealand) and LabChart 8 Pro software (ADInstruments, Colorado Springs, CO, USA). ECG parameters, namely, the cardiac cycle length, RR, QRS, PQ, QT, corrected QTc, JT intervals, T wave amplitude, and HRV were recorded using 5-lead electrocardiography.

HRV was calculated using the time and frequency domains. Each HRV analysis was performed using two-minute intervals and assigned to the middle point of this interval. Three frequency power components in the frequency domain of HRV were calculated: VLF at frequencies ranging from 0–0.01 Hz, LF at frequencies ranging from 0.01 Hz–0.09 Hz, and HF at frequencies ranging from 0.09 Hz–2 Hz, and all these components were reported as a ratio with total power of frequency (Tot P). For the time domain of HRV beat-to-beat analyses, the standard deviation of RR intervals (SDRR), standard deviation of successive RR interval differences (SDSD), and root mean square of successive differences (RMSSD) were obtained. All HRV parameters were determined using the LabChart 8 Pro software HRV module (ADInstruments, Colorado Springs, CO, USA).

### 4.6. Plasma Preparation for the Pharmacokinetic Analysis

Blood samples (1–1.5 mL) were collected into anticoagulant EDTA-treated tubes and maintained on ice during the experiment until centrifugation. Cells and platelets were removed from plasma by centrifugation for 15 min at 2000× *g* (10 °C). The supernatant (plasma) was immediately collected into a polypropylene tube using a single-channel pipette, and the plasma samples were maintained on ice during handling. Plasma aliquots of 0.2 mL were stored at −80 °C until the mass spectrometry analysis.

### 4.7. Ultraperformance Liquid Chromatography–Electrospray Ionization–Tandem Mass Spectrometry (UPLC–ESI–MS/MS) Analysis of Plasma Samples

Chromatographic separation of plasma samples was performed on an Aquity H–class UPLC system (Waters, Milford, MA, USA). A BEH C18 (100 × 2.1 mm 1.7 µm) column was used for the separation of ketone compounds and their metabolites. The column temperature was maintained at 40 °C. Gradient elution was performed using a mobile phase consisting of 0.1% formic acid water solution (solvent A) and acetonitrile (solvent B) with the flow rate set to 0.5 mL/min. A linear gradient profile was applied with the following proportions of solvent A: 0 to 0.5 min, 95%; 3.5 min, 5%; 3.6 min, and back to 95% for a total analysis time of 5 min. The MS/MS analysis of the separated peaks of compounds was performed with a triple quadrupole tandem mass spectrometer (Xevo, Waters, Milford, MA, USA). Electrospray ionization was applied in positive mode for analysis with the following settings: capillary voltage, 4 kV; source temperature, 150 °C; desolvation temperature, 600 °C; desolvation gas flow, 650 L/h; and cone gas flow, 50 L/h. IntelliStart software (Waters, Milford, MA, USA) was used to develop a specific collision energy and cone voltage for each compound of interest. Analytical grade standards purchased from ChemSpace (Riga, Latvia), EK ketone (3-methyl-1-(3-methylfuran-2-yl) butan-1-one from ChemFaces (Wuhan, China), and DEK—*Naginata* ketone were used for quantitative analysis of DEK and EK concentrations.

### 4.8. Blood Parameters

The following haematological parameters were measured: WBCs (10^9^ cells L^−1^), lymphocytes (LYM, % and 10^9^ cells L^−1^), monocytes (MON, % and 10^9^ cells L^−1^), granulocytes (GRA, % and 10^9^ cells L^−1^), red blood cells (RBCs, 10^12^ cells L^−1^), haemoglobin (HGB, g/L), haematocrit (HCT, %), mean corpuscular volume (MCV, Fl), mean corpuscular haemoglobin (MCH, pg), mean corpuscular haemoglobin concentration (MCHC, g/L), red cell distribution width (RDW, %), PLT (10^9^ platelets L^−1^), mean platelet volume (MPV, Fl), procalcitonin (PCT, %), and platelet distribution width (PDW, %). An EPOC blood gas analyser was used to measure the concentrations of the following molecules in serum: sodium (Na^+^, mmol/L), potassium (K^+^, mmol/L), calcium (Ca^2+^, mmol/L), chlorine (Cl^−^, mmol/L), glucose (Glu, mmol/L), lactate (Lac, mmol/L), blood urea nitrogen (BUN, mg/dL), creatinine (Crea, mg/dL), and pH. A venous blood gas analysis was performed to assess partial oxygen pressure (PO, mmHg), partial carbon dioxide pressure (PCO_2_, mmHg), counted oxygen saturation (cSO_2_, %), counted bicarbonate (cHCO^−3^, mmol/L), blood total carbon dioxide (CTCO_2_, mmHg), and base excess (BE, mmol/L).

### 4.9. Statistical Analysis

Data are presented as the means ± standard deviation (SD). The significance of differences between the control group and treatment group at various time points was evaluated using a paired and two-tailed distribution *t* test in MS Excel 2010 (Microsoft, Brussels, Belgium). A *p* value < 0.05 was considered statistically significant. Before the *t* test, data was assessed for normality using the Jarque-Bera normality test and Levene’s test for homogeneity of variance was performed using the Real Statistics Resource Pack for Excel. In all cases, Levene’s test was statistically insignificant (*p* > 0.05), allowing us to use a paired *t* test. 

During the analysis of the data from each experiment, logistic sigmoidal curves were constructed to approximate blood pressure and HR and to calculate t_1/2_ from the curves. The t_1/2_ of the curves showing increased HR were compared with the t_1/2_ of the sigmoidal curves showing decreases in arterial blood pressure to assess their onset of action. The increase in HR had two phases. The first component of the increase in HR was not observed in all experiments; however, in 4 experiments, it was sufficiently large to estimate the data by approximation with the sigmoidal curve using MS Excel 2010 (Microsoft, Brussels, Belgium). Mathematical subtraction of the first component of the increased HR was performed by approximating it with an asymmetric Gaussian curve to calculate the second phase of the increase in HR. 

The average data for DEK metabolites were normalized to their maximum values and approximated using the Hill equation for the time response function. The correlation coefficients (R^2^) characterizing the quality of fit of the Hill equation with the experimental data were calculated using MS Excel 2010 (Microsoft, Brussels, Belgium).

## 5. Conclusions

The results of the study confirm that *E. ciliata* possesses properties similar to those of class 1B antiarrhythmics and exerts a hypotensive effect by reducing arterial blood pressure through effects on peripheral vascular resistance.

This dual-acting effect of an agent such as *EO* may be effective for patients with cardiac arrhythmias and hypertension. Due to the increasing number of patients diagnosed with cardiovascular diseases, medicines that have dual activity may be particularly valuable.

Alterations in the autonomic nervous system are the response of an organism to the hypotensive effect of an *EO* bolus to increase blood pressure.

In this study, we analysed the short-term effects of an intravenous EO injection. The investigation of other EO administration methods that might prolong the effects of the herbal extract would be useful.

## Data Availability

Data is contained within the article.

## Supplementary Materials

The following supporting information can be downloaded at: https://www.mdpi.com/article/10.3390/ph15080982/s1, Figure S1: Effect of an intravenous bolus of EO on cardiac electrical activity in swine. Changes in electrocardiogram parameters over time: cardiac cycle ECG recordings at different times after EC bolus: RR interval (a); T wave amplitude (b); and QT interval (c). The data are expressed as the percent change compared to the control. * *p* < 0.05 indicates a significant difference from controls, *n* = 8; Table S1: Chemical composition of essential oil obtained by hydrodistillation from *E. ciliata* dried (2021 August) herb; Table S2: Time domain of HRV changes after an intravenous bolus of *EC* in swine. * *p* < 0.05 indicates a significant difference compared to the control, *n* = 8; Table S3: Blood parameter changes after an intravenous *EO* bolus in swine at different time points. Mean ± SD. * *p* < 0.05 indicates a significant difference compared to the control, *n* = 8.

## References

[1] Shenasa M., Shenasa H. (2017). Hypertension, Left Ventricular Hypertrophy, and Sudden Cardiac Death. Int. J. Cardiol..

[2] Srinivasan N.T., Schilling R.J. (2018). Sudden Cardiac Death and Arrhythmias. Arrhythmia Electrophysiol. Rev..

[3] Schiweck C., Piette D., Berckmans D., Claes S., Vrieze E. (2019). Heart Rate and High Frequency Heart Rate Variability during Stress as Biomarker for Clinical Depression. A Systematic Review. Psychol. Med..

[4] Darbar D., Roden D.M. (2006). Future of Antiarrhythmic Drugs. Curr. Opin. Cardiol..

[5] Godfraind T. (2017). Discovery and Development of Calcium Channel Blockers. Front. Pharmacol..

[6] Andersen S.S., Hansen M.L., Gislason G.H., Schramm T.K., Folke F., Fosbol E., Abildstrom S.Z., Madsen M., Kober L., Torp-Pedersen C. (2009). Antiarrhythmic Therapy and Risk of Death in Patients with Atrial Fibrillation: A Nationwide Study. Europace.

[7] Malhotra S., Das M.K. (2011). Delayed and Indirect Effects of Antiarrhythmic Drugs in Reducing Sudden Cardiac Death. Future Cardiol..

[8] Camm A.J. (2017). Hopes and Disappointments with Antiarrhythmic Drugs. Int. J. Cardiol..

[9] D’Souza S.P., Chavannavar S.V., Kanchanashri B., Niveditha S.B. (2017). Pharmaceutical Perspectives of Spices and Condiments as Alternative Antimicrobial Remedy. J. Evid.-Based Complementary Altern. Med..

[10] Tongnuanchan P., Benjakul S. (2014). Essential Oils: Extraction, Bioactivities, and Their Uses for Food Preservation. J. Food Sci..

[11] Wińska K., Mączka W., Łyczko J., Grabarczyk M., Czubaszek A., Szumny A. (2019). Essential Oils as Antimicrobial Agents—Myth or Real Alternative?. Molecules.

[12] Aguiar F.C., Solarte A.L., Gómez-Gascón L., Galán-Relaño A., Luque I., Tarradas C., Rodríguez-Ortega M.J., Huerta B. (2022). Antimicrobial Susceptibility of Cinnamon and Red and Common Thyme Essential Oils and Their Main Constituent Compounds against *Streptococcus suis*. Lett. Appl. Microbiol..

[13] Fratini F., Forzan M., Turchi B., Mancini S., Alcamo G., Pedonese F., Pistelli L., Najar B., Mazzei M. (2020). In Vitro Antibacterial Activity of Manuka (*Leptospermum scoparium* J.R. et G. Forst) and Winter Savory (*Satureja montana* L.) Essential Oils and Their Blends against Pathogenic *E. coli* Isolates from Pigs. Animals.

[14] Sharifi-Rad J., Soufi L., Ayatollahi S.A.M., Iriti M., Sharifi-Rad M., Varoni E.M., Shahri F., Esposito S., Kuhestani K., Sharifi-Rad M. (2016). Anti-Bacterial Effect of Essential Oil from Xanthium Strumarium against Shiga Toxin-Producing *Escherichia coli*. Cell Mol. Biol..

[15] Wu Z., Tan B., Liu Y., Dunn J., Martorell Guerola P., Tortajada M., Cao Z., Ji P. (2019). Chemical Composition and Antioxidant Properties of Essential Oils from Peppermint, Native Spearmint and Scotch Spearmint. Molecules.

[16] Aljeldah M.M. (2022). Antioxidant and Antimicrobial Potencies of Chemically-Profiled Essential Oil from *Asteriscus graveolens* against Clinically-Important Pathogenic Microbial Strains. Molecules.

[17] Cardia G.F.E., Silva-Comar F.M.D.S., Silva E.L., da Rocha E.M.T., Comar J.F., Silva-Filho S.E., Zagotto M., Uchida N.S., Bersani-Amado C.A., Cuman R.K.N. (2021). Lavender (*Lavandula officinalis*) essential oil prevents acetaminophen-induced hepatotoxicity by decreasing oxidative stress and inflammatory response. Res. Soc. Dev..

[18] Brochot A., Guilbot A., Haddioui L., Roques C. (2017). Antibacterial, Antifungal, and Antiviral Effects of Three Essential Oil Blends. Microbiologyopen.

[19] Okano S., Honda Y., Kodama T., Kimura M. (2019). The Effects of Frankincense Essential Oil on Stress in Rats. J. Oleo Sci..

[20] Pereira G.L.D.C., Almeida T.C., Seibert J.B., Amparo T.R., Soares R.D.D.O.A., Rodrigues I.V., de Souza G.H.B., dos Santos O.D.H., da Silva G.N. (2021). Antitumor Effect of *Cymbopogon densiflorus* (Linneu) Essential Oil in Bladder Cancer Cells. Nat. Prod. Res..

[21] Lee G., Park J., Kim M.S., Seol G.H., Min S.S. (2019). Analgesic Effects of Eucalyptus Essential Oil in Mice. Korean J. Pain.

[22] Labib R.M., Ayoub I.M., Michel H.E., Mehanny M., Kamil V., Hany M., Magdy M., Moataz A., Maged B., Mohamed A. (2019). Appraisal on the Wound Healing Potential of Melaleuca Alternifolia and *Rosmarinus officinalis* L. Essential Oil-Loaded Chitosan Topical Preparations. PLoS ONE.

[23] Guo Z., Liu Z., Wang X., Liu W., Jiang R., Cheng R., She G. (2012). Elsholtzia: Phytochemistry and Biological Activities. Chem. Cent. J..

[24] Tian G. (2014). Chemical Constituents in Essential Oils from *Elsholtzia ciliata* and Their Antimicrobial Activities. Chin. Herb. Med..

[25] Pudziuvelyte L., Stankevicius M., Maruska A., Petrikaite V., Ragazinskiene O., Draksiene G., Bernatoniene J. (2017). Chemical Composition and Anticancer Activity of *Elsholtzia ciliata* Essential Oils and Extracts Prepared by Different Methods. Ind. Crops Prod..

[26] Mačianskienė R., Pudžiuvelytė L., Bernatonienė J., Almanaitytė M., Navalinskas A., Treinys R., Andriulė I., Jurevičius J. (2020). Antiarrhythmic Properties of *Elsholtzia ciliata* Essential Oil on Electrical Activity of the Isolated Rabbit Heart and Preferential Inhibition of Sodium Conductance. Biomolecules.

[27] Bernatoniene J., Pudziuvelyte L., Jurevicius J., Macianskiene R., Simonyte S. (2021). Elsholtzia Ciliata Essential Oil Extract as Antiarrhythmic Drug. WO Patent.

[28] Van Essen G.J., Hekkert M.T.L., Sorop O., Heinonen I., van der Velden J., Merkus D., Duncker D.J. (2018). Cardiovascular Function of Modern Pigs Does Not Comply with Allometric Scaling Laws. Sci. Rep..

[29] Muzykantov V.R. (2010). Drug Delivery by Red Blood Cells: Vascular Carriers Designed by Mother Nature. Expert Opin. Drug Deliv..

[30] Romagnoli N., Al-Qudah K.M., Armorini S., Lambertini C., Zaghini A., Spadari A., Roncada P. (2017). Pharmacokinetic Profile and Partitioning in Red Blood Cells of Romifidine after Single Intravenous Administration in the Horse. Vet. Med. Sci..

[31] Tian M., Peng Y., Zheng J. (2022). Metabolic Activation and Hepatotoxicity of Furan-Containing Compounds. Drug Metab. Dispos..

[32] Adams P. (2019). Identification of Essential Oil Components by Gas Chromatography/Mass Spectrometry.

[33] Pingzhao M., Chaoliu X., Lai D., Zhou L., Longliu Z. (2016). Analysis of the Essential Oil of Elsholtzia Ciliate Aerial Parts and Its Insecticidal Activities against *Liposcelis bostrychophila*. Helv. Chim. Acta.

[34] de Boer R.W., Karemaker J.M. (2019). Cross-Wavelet Time-Frequency Analysis Reveals Sympathetic Contribution to Baroreflex Sensitivity as Cause of Variable Phase Delay between Blood Pressure and Heart Rate. Front. Neurosci..

[35] Bertram D., Barrès C., Cuisinaud G., Julien C. (1998). The Arterial Baroreceptor Reflex of the Rat Exhibits Positive Feedback Properties at the Frequency of Mayer Waves. J. Physiol..

[36] Gulli G., Cooper V.L., Claydon V.E., Hainsworth R. (2005). Prolonged Latency in the Baroreflex Mediated Vascular Resistance Response in Subjects with Postural Related Syncope. Clin. Auton. Res..

[37] Von Borell E., Langbein J., Després G., Hansen S., Leterrier C., Marchant-Forde J., Marchant-Forde R., Minero M., Mohr E., Prunier A. (2007). Heart Rate Variability as a Measure of Autonomic Regulation of Cardiac Activity for Assessing Stress and Welfare in Farm Animals—A Review. Physiol. Behav..

[38] Shafi T., Mullangi S., Jaar B.G., Silber H. (2017). Autonomic Dysfunction as a Mechanism of Intradialytic Blood Pressure Instability. Semin. Dial..

[39] Gabor A., Leenen F.H. (2012). Mechanisms of Sympathetic Regulation in Cardiovascular Disease Central Neuromodulatory Pathways Regulating Sympathetic Activity in Hypertension. J. Appl. Physiol..

[40] Byrd C.J., Johnson J.S., Radcliffe J.S., Craig B.A., Eicher S.D., Lay D.C. (2020). Nonlinear Analysis of Heart Rate Variability for Evaluating the Growing Pig Stress Response to an Acute Heat Episode. Animal.

[41] Robertson D., Biaggioni I., Burnstock G., Low A.P., Paton F.R.J. (2012). Primer on the Autonomic Nervous System.

[42] Shaffer F., Ginsberg J.P. (2017). An Overview of Heart Rate Variability Metrics and Norms. Front. Public Health.

[43] Armstrong M., Kerndt C.C., Moore R.A. (2022). Physiology, Baroreceptors.

[44] Bonyhay I., Freeman R. (2004). Sympathetic Nerve Activity in Response to Hypotensive Stress in the Postural Tachycardia Syndrome. Circulation.

[45] Poletto R., Janczak A.M., Marchant-Forde R.M., Marchant-Forde J.N., Matthews D.L., Dowell C.A., Hogan D.F., Freeman L.J., Lay D.C. (2011). Identification of Low and High Frequency Ranges for Heart Rate Variability and Blood Pressure Variability Analyses Using Pharmacological Autonomic Blockade with Atropine and Propranolol in Swine. Physiol. Behav..

[46] Del Colle S., Morello F., Rabbia F., Milan A., Naso D., Puglisi E., Mulatero P., Veglio F. (2007). Antihypertensive Drugs and the Sympathetic Nervous System. J. Cardiovasc. Pharmacol..

[47] Grassi G. (2016). Sympathomodulatory Effects of Antihypertensive Drug Treatment. Am. J. Hypertens..

[48] Katsurada K., Kario K. (2021). Sympathetic Modulation by Antihypertensive Drugs. J. Clin. Hypertens..

[49] de Champlain J., Karas M., Toal C., Nadeau R., Larochelle P. (1999). Effects of Antihypertensive Therapies on the Sympathetic Nervous System. Can. J. Cardiol..

[50] Lefrant J.-Y., Muller L., de La Coussaye J.E., Lalourcey L., Ripart J., Peray P.A., Mazoit X., Dauzat M., Sassine A., Eledjam J.-J. (2003). Hemodynamic and Cardiac Electrophysiologic Effects of Lidocaine–Bupivacaine Mixture in Anesthetized and Ventilated Piglets. Anesthesiology.

[51] McKeever R.G., Hamilton R.J. (2022). Calcium Channel Blockers.

[52] Freed M.I., Rastegar A., Bia M.J. (1191). Effects of Calcium Channel Blockers on Potassium Homeostasis. Yale J. Biol. Med..

[53] Somers M.P., Brady W.J., Perron A.D., Mattu A. (2002). The Prominant T Wave: Electrocardiographic Differential Diagnosis. Am. J. Emerg. Med..

[54] Manoury B., Idres S., Leblais V., Fischmeister R. (2020). Ion Channels as Effectors of Cyclic Nucleotide Pathways: Functional Relevance for Arterial Tone Regulation. Pharmacol. Ther..

[55] Yanagisawa T., Yamagishi T., Okada Y. (1993). Hyperpolarization Induced by K^+^ Channel Openers Inhibits Ca^2+^ Influx and Ca^2+^ Release in Coronary Artery. Cardiovasc. Drugs Ther..

[56] de Simone G., Pasanisi F. (2001). Systolic, Diastolic and Pulse Pressure: Pathophysiology. Ital. Heart J. Suppl..

[57] King D.R., Entz M., Blair G.A., Crandell I., Hanlon A.L., Lin J., Hoeker G.S., Poelzing S. (2021). The Conduction Velocity-Potassium Relationship in the Heart Is Modulated by Sodium and Calcium. Pflüg. Arch. Eur. J. Physiol..

[58] Pudziuvelyte L., Liaudanskas M., Jekabsone A., Sadauskiene I., Bernatoniene J. (2020). *Elsholtzia ciliata* (Thunb.) Hyl. Extracts from Different Plant Parts: Phenolic Composition, Antioxidant, and Anti-Inflammatory Activities. Molecules.

[59] Chatterjee A., Dutta U., Bandyopadhyay D., Nayak A., Basak B., Banerji A., Banerji J. (2007). An Overview of the Genus Nardostachys. Nat. Prod. Commun..

[60] Roullet J.-B., Luft U.C., Xue H., Chapman J., Bychkov R., Roullet C.M., Luft F.C., Haller H., McCarron D.A. (1997). Farnesol Inhibits L-Type Ca^2+^ Channels in Vascular Smooth Muscle Cells. J. Biol. Chem..

[61] Gonçalves M.S.S., Barreto A.S. (2021). Nerolidol, a Sesquiterpene Alcohol, Attenuates Acute Myocardial Infarction in Rats.

[62] du Sert N.P., Hurst V., Ahluwalia A., Alam S., Avey M.T., Browne W.J., Clark A. (2020). The ARRIVE Guidelines 2.0: Updated Guidelines for Reporting Animal Research. PLoS Biol..

