# Supplementary material for: Evaluation of the Cardiac Electrophysiological and Haemodynamic Effects of Elsholtzia ciliata Essential Oil on Swine
Source: Pharmaceuticals (Basel). 2022 Aug 10;15(8):982. doi: 10.3390/ph15080982 (PMC9414655; doi:10.3390/ph15080982)
Supplement: Supplementary file 1 [file pharmaceuticals-15-00982-s001.zip › pharmaceuticals-1744037-supplementary.pdf]

**Supplementary Material**

**Table S1.** Chemical composition of essential oil obtained by hydrodistillation from *E. ciliata* dried (2021 August) herb.

| **Compounds** | ***RI$_{calculated}$** | ***RI$_{theoretical}$** | **Composition, %** |
|---|---|---|---|
| 2-Ethylfuran | 702 | 721 | 0.05 |
| 2-acetyl-5-methylfuran | 972 | 965 | 0.06 |
| Eucalyptol | 963 | 954 | 0.07 |
| Rosefuran | 1023 | 1012 | 0.08 |
| Elsholtzia ketone | 1066 | 1045 | 14.12 |
| Furane-2-carboxaldehyde. 5-(nitrophenoxymethyl)- | 1079 | 1057 | 0.37 |
| (-)-1R-8-Hydroxy-p-menth-4-en-3-one | 1110 | 1098 | 0.07 |
| Dehydroelsholtzia ketone | 1117 | 1095 | 78.15 |
| Eugenol | 1140 | 1107 | 0.15 |
| Beta-Bourbonene | 1152 | 1121 | 0.66 |
| Isocaryophyllene | 1166 | 1134 | 0.62 |
| Beta-Cubebene | 1170 | 1147 | 0.09 |
| Ledene | 1174 | 1177 | 0.07 |
| Alpha-Caryophyllene | 1180 | 1176 | 1.87 |
| Alpha-Cubebene | 1186 | 1184 | 0.05 |
| Naphthalene | 1190 | 1188 | 0.12 |
| Germacrene D | 1192 | 1194 | 0.28 |
| Trans-alpha-Bergamotene | 1197 | 1191 | 0.67 |
| Alpha-Farnesene | 1202 | 1194 | 0.75 |
| Gamma-Cadinene | 1205 | 1189 | 0.18 |
| Delta-Cadinene | 1208 | 1199 | 0.33 |
| Caryophyllene oxide | 1224 | 1203 | 0.24 |
| Nonane | 1243 | 1212 | 0.06 |
| Palmitic acid | 1275 | 1254 | 0.14 |
| Phytol | 1286 | 1273 | 0.08 |
| Methyl (Z)-5.11.14.17-eicosatetraenoate | 1289 | 1279 | 0.58 |
| 2.6-octadiene. 2.7-dimethyl- | 1294 | 1288 | 0.09 |
| Sesquiterpenes | - | - | 5.93 |
| Oxygenated monoterpenes | - | - | 0.22 |
| Oxygenated sesquiterpenes | - | - | 5.69 |
| Ketones | - | - | 92.27 |
| Others | - | - | 1.58 |
| Total | - | - | 100.0 |

*RI – Retention Index

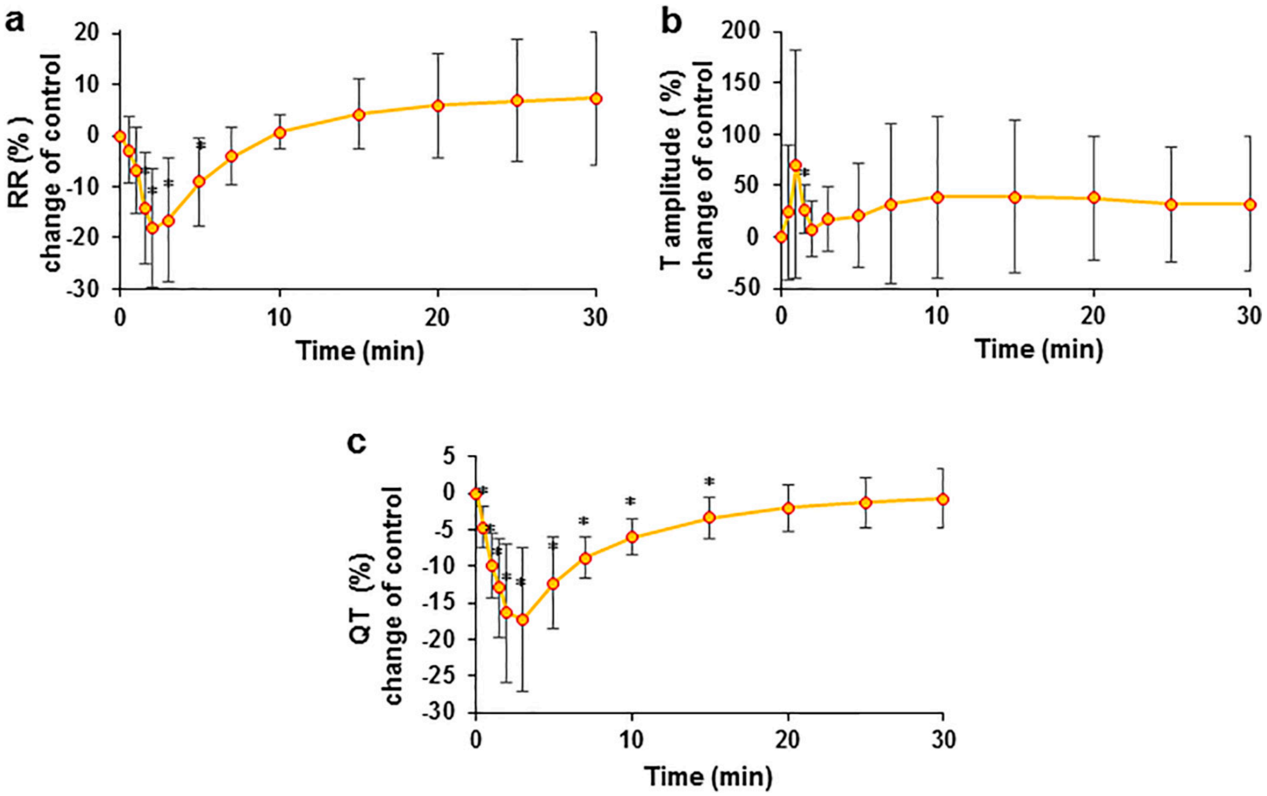


**Figure S1.** Effect of an intravenous bolus of EO on cardiac electrical activity in swine. Changes in electrocardiogram parameters over time: cardiac cycle ECG recordings at different times after EC bolus: RR interval (a); T wave amplitude (b); and QT interval (c). The data are expressed as the percent change compared to the control. * $p < 0.05$ indicates a significant difference from controls, n = 8.

**Table S2.** Time domain of HRV changes after an intravenous bolus of *EC* in swine. * $p < 0.05$ indicates a significant difference compared to the control, n = 8.

| Parameter | Control | T 2 | T 5 | T 10 | T 20 | T 30 |
|---|---|---|---|---|---|---|
| SDRR | 3.15 ±1.48 | 28.47* ±22.58 | 13.2* ±8.71 | 5.88 ±3.87 | 4.55* ±2.01 | 4.01 ±1.78 |
| SDSD | 2.92 ±1.31 | 3.85 ±3.77 | 4.76 ±6.52 | 3.87 ±2.98 | 3.49 ±2.29 | 3.31 ±1.677 |
| RMSSD | 2.91 ±1.30 | 3.88 ±3.76 | 4.76 ±6.50 | 3.86 ±2.97 | 3.49 ±2.29 | 3.30 ±1.66 |

**Table S3.** Blood parameter changes after an intravenous *EO* bolus in swine at different time points. Mean ± SD. * $p < 0.05$ indicates a significant difference compared to the control, n = 8.

| Parameter | T 0 | | T 1 | | T 5 | | T 30 | |
|---|---|---|---|---|---|---|---|---|
| WBC | 12.24 | 2.47 | 10.23* | 2.11 | 10.38* | 2.82 | 11.44 | 2.92 |
| LYM | 6.80 | 1.59 | 5.94* | 1.52 | 6.05* | 1.45 | 6.44 | 1.67 |
| MON | 0.38 | 0.15 | 0.30* | 0.12 | 0.26 | 0.09 | 0.26* | 0.09 |
| NEU | 5.08 | 2.31 | 3.98* | 1.82 | 4.04 | 2.11 | 4.70 | 2.59 |
| RBC | 5.88 | 1.07 | 5.84 | 1.11 | 5.95 | 1.19 | 5.79 | 1.19 |
| HGB | 109.75 | 22.11 | 107.63 | 22.58 | 109.00 | 23.29 | 107.25 | 25.26 |
| HCT | 0.36 | 0.08 | 0.36 | 0.09 | 0.38 | 0.08 | 0.37 | 0.08 |
| MCV | 61.46 | 7.49 | 61.59 | 7.89 | 60.36 | 10.11 | 63.48 | 2.70 |
| MCH | 18.73 | 0.61 | 18.38 | 0.58 | 18.28 | 0.84 | 18.41 | 0.76 |
| MCHC | 309.13 | 55.32 | 304.88 | 53.52 | 287.13 | 16.11 | 290.25 | 13.83 |
| RDW | 15.68 | 3.10 | 15.41 | 3.24 | 15.11 | 1.37 | 14.91 | 1.44 |
| PLT | 318.25 | 75.97 | 290.38* | 76.02 | 289.75 | 106.49 | 317.75 | 59.53 |
| MPV | 7.66 | 0.65 | 7.59 | 0.59 | 7.54 | 0.32 | 7.66 | 0.43 |
| PCT | 0.24 | 0.05 | 0.22* | 0.05 | 0.22 | 0.08 | 0.24 | 0.04 |
| PDW | 17.81 | 2.02 | 18.91 | 2.82 | 17.54 | 4.14 | 18.99 | 1.42 |
| pH | 7.51 | 0.09 | 7.52 | 0.07 | 7.54 | 0.08 | 7.50 | 0.10 |
| $pCO_2$ | 40.39 | 10.24 | 37.93 | 5.99 | 35.85 | 7.75 | 39.81 | 11.02 |
| $pO_2$ | 48.64 | 13.00 | 54.78 | 11.48 | 54.69 | 13.00 | 45.76 | 8.52 |
| $cHCO^{3-}$ | 31.15 | 3.83 | 30.79 | 3.51 | 30.05* | 3.47 | 29.93 | 3.71 |
| $cSO_2$ | 83.34 | 10.79 | 89.39 | 5.69 | 88.74* | 8.80 | 81.88 | 11.91 |
| $CTCO_2$ | 30.40 | 3.67 | 30.00 | 3.32 | 29.29* | 3.28 | 29.28 | 3.56 |
| BE (b) | 7.54 | 3.34 | 7.39 | 3.56 | 7.21 | 3.35 | 6.35 | 3.18 |
| BE (ecf) | 8.03 | 3.80 | 7.93 | 3.95 | 7.48* | 3.77 | 6.70 | 3.66 |
| $Na^+$ | 142.88 | 1.73 | 140.63 | 3.93 | 141.50 | 1.77 | 141.50 | 2.00 |
| $K^+$ | 3.70 | 0.34 | 3.88* | 0.44 | 3.90* | 0.42 | 3.93* | 0.44 |
| $Ca^{2+}$ | 1.22 | 0.09 | 1.16 | 0.13 | 1.19 | 0.11 | 1.21 | 0.10 |
| $Cl^-$ | 102.88 | 2.42 | 103.63 | 2.72 | 103.13 | 2.75 | 102.25 | 2.38 |
| Hct | 31.00 | 6.76 | 31.38 | 7.73 | 31.63 | 7.61 | 30.75 | 7.15 |
| cHgb | 10.58 | 2.32 | 10.69 | 2.61 | 9.45 | 4.18 | 10.45 | 2.47 |
| Glu | 55.38 | 20.58 | 55.63 | 22.60 | 63.25 | 31.24 | 72.63 | 37.80 |
| Lac | 1.73 | 0.54 | 1.81 | 0.41 | 1.93 | 0.45 | 2.56 | 0.93 |
| BUN | 5.38 | 2.07 | 5.50 | 1.77 | 5.13 | 1.13 | 5.50 | 2.00 |
| Crea | 1.39 | 0.30 | 1.42 | 0.29 | 1.37 | 0.27 | 1.49* | 0.32 |
